# Light induced intraspecific variability in response to thermal stress in the hard coral *Stylophora pistillata*

**DOI:** 10.7717/peerj.3802

**Published:** 2017-10-11

**Authors:** Arjen Tilstra, Tim Wijgerde, Francisco Dini-Andreote, Britas Klemens Eriksson, Joana Falcão Salles, Ido Pen, Ronald Osinga, Christian Wild

**Affiliations:** 1 Department of Marine Benthic Ecology & Evolution, Groningen Institute for Evolutionary Life Sciences, University of Groningen, Groningen, The Netherlands; 2 Marine Ecology Group, Faculty of Biology and Chemistry, University of Bremen, Bremen, Germany; 3 Coral Publications, Utrecht, The Netherlands; 4 Microbial Ecology cluster, Groningen Institute for Evolutionary Life Sciences, University of Groningen, Groningen, The Netherlands; 5 Theoretical Research in Evolutionary Life Sciences, Groningen Institute for Evolutionary Life Sciences, University of Groningen, Groningen, The Netherlands; 6 Marine Animal Ecology Group, Wageningen University & Research, Wageningen, The Netherlands

**Keywords:** *Stylophora pistillata*, Thermal tolerance, Light stress, Necrosis, Photoprotection, Bleaching, Intraspecific variability

## Abstract

Recent research suggests that prior exposure of several months to elevated irradiance induces enhanced thermal tolerance in scleractinian corals. While this tolerance has been reported at the species level, individual coral colonies may react differently due to individual variability in thermal tolerance. As thermal anomalies are predicted to become common in the upcoming future, intraspecific variation may be key to the survival of coral populations. In order to study light-history based thermal stress responses on individual colonies, we developed a preliminary microcosm experiment where three randomly chosen, aquacultured colonies of the model coral *Stylophora pistillata* were exposed to two irradiance treatments (200 and 400 μmol photons m^−2^ s^−1^) for 31 days, followed by artificially induced heat stress (∼33.4 °C). We found different responses to occur at both the intraspecific and the intracolonial levels, as indicated by either equal, less severe, delayed, and/or even non-necrotic responses of corals previously exposed to the irradiance of 400 compared to 200 μmol photons m^−2^ s^−1^. In addition, all individual colonies revealed light-enhanced calcification. Finally, elevated irradiance resulted in a lower chlorophyll *a* concentration in one colony compared to the control treatment, and the same colony displayed more rapid bleaching compared to the other ones. Taken together, this study highlights the potential importance of intra-individual variability in physiological responses of scleractinian corals and provides recommendations for improving methodological designs for future studies.

## Introduction

Light plays an integral part in the life cycle of many scleractinian corals due to their symbiotic relationship with photosynthetic, unicellular dinoflagellates belonging to the genus *Symbiodinium*. These symbionts (commonly referred to as zooxanthellae) provide the coral host with photosynthetically derived metabolic energy (Muscatine, 1973; Muscatine & Porter, 1977; Trench, 1979; Falkowski et al., 1984; Edmunds & Davies, 1986; Muller-Parker & D’Elia, 1997) and are of extreme importance for the physiological status of the coral. Prolonged exposure to elevated solar irradiance (Brown et al., 1994; Le Tissier & Brown, 1996), thermal anomalies (Hoegh-Guldberg, 1999), or both (Brown, 1997; Hawkins et al., 2015) are known to compromise the symbiotic relationship between corals and zooxanthellae. The outcome is the progressive dissociation of the coral and its symbionts and/or their photopigments, a phenomenon known as bleaching (Hoegh-Guldberg & Smith, 1989). Under severe thermal stress, corals may also express tissue loss/necrosis (Schoepf et al., 2015), which can ultimately lead to organismal death. However, exposure to elevated solar irradiance (over several months) prior to thermal stress has been recently reported to alter thermal tolerance of corals (Brown et al., 2000a, 2002a, 2002b). These authors showed that west-facing surfaces of the scleractinian coral *Coelastrea aspera* (formerly known as *Goniastrea aspera*; Huang et al., 2014) exposed to elevated irradiance in the PAR region (photosynthetically active radiation, 400–700 nm) exhibited elevated tolerance to thermally induced bleaching comparatively to east-facing surfaces of the same colonies. This was evidenced by bleaching on the east-facing surfaces, while west-facing surfaces retained pigmentation during a thermal stress event. Interestingly, the effect of irradiance enhanced thermal tolerance (IETT) was reported to be retained for at least 10 years (Brown et al., 2015). Moreover, the literature has shown that this phenomenon is not mediated by differences in the zooxanthellae-associated community (Brown et al., 2002a, 2002b), but rather by coral host physiology via increasing expression of antioxidizing agents and stress-related proteins (Brown et al., 2002b).

Reefs exposed to thermal stress often contain conspecifics that display both bleached and unbleached phenotypes (Sotka & Thacker, 2005). This heterogeneity in thermal response can be related to the genotype (clades or types) of *Symbiodinium* associated with specific coral colonies (Sotka & Thacker, 2005), thermal tolerance of the coral host (Bellantuono, Hoegh-Guldberg & Rodriguez-Lanetty, 2012; Shaw et al., 2016), and possibly the associated bacterial community (Ziegler et al., 2017). While interspecific variation in response to thermal stress, particularly in hard corals, is well described in the literature, information on intraspecific and intracolonial variation remains elusive (Edmunds, 1994; Brown et al., 2002a; Pratchett et al., 2013). Hence, the organismal level variability in thermal stress susceptibility may be key to the survival of coral reefs. Thus, as bleaching events are predicted to become common in the upcoming future (Hoegh-Guldberg et al., 2007; van Hooidonk et al., 2016), understanding the symbiotic relationship between corals and zooxanthellae within a single species or colony during light exposure and thermal stress is conceivable of major ecological and conservational interests.

*Coelastrea* is known to be less susceptible to bleaching (Baird et al., 2009) when compared to, e.g., the more commonly used model organism *Stylophora pistillata* (McClanahan et al., 2004). In addition, *S. pistillata* has strong intraspecific differences in physiological (stress) properties (Downs et al., 2009; Osinga et al., 2012). The present study aims at exploring (i) whether the hard coral *Stylophora pistillata* (Esper, 1797) displays intraspecific variability in thermal stress responses after pre-exposure to an elevated light treatment; (ii) whether the coral colonies can obtain IETT after the pre-exposure to elevated light; and (iii) whether pooled data of the conspecific individuals yielded similar results compared to the individual coral colonies. Colonies of *S. pistillata* were exposed to two irradiance levels, i.e., 200 or 400 μmol photons m^−2^ s^−1^ respectively, and subsequently to artificially induced heat stress. We examined several characteristics of the coral host as well as the dinoflagellate symbiont over time, i.e., specific growth rate (SGR), the occurrence of bleaching and/or necrosis, survival, zooxanthellae cell density, chlorophyll *a* concentration, photosynthetic efficiency (effective quantum yield (EQY) of photosystem II (PSII)), and zooxanthellae community composition.

## Materials and Methods

### Sample species and experimental design

**Figure 1:**
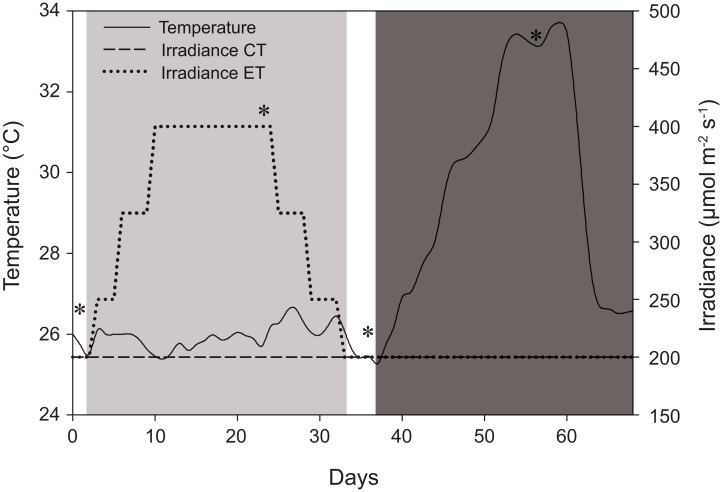
Schematic representation of the experimental design displaying variations in light and temperature over the 68-day interval. The solid line represents temperature (for both light treatments) in °C. The dashed line, control treatment (CT), and dotted line, experimental treatment (ET), represent light intensity (irradiance) in μmol m^−2^ s^−1^. *Days of sampling (i.e., at days 1, 24, 36, and 56). Day 2–33: light treatment (light gray background); day 37–68: heat stress (dark gray background).

Three parent colonies of the scleractinian coral species *S. pistillata* were randomly chosen from a large pool of colonies resulting in two morphotypes and three color variations. Colony 1 (C1) displayed a bright pink phenotype with thick branches and was collected in Indonesia (T Hoff, 2016, personal communication), thus belonging to clade 1 (Keshavmurthy et al., 2013). Colony 2 (C2) displayed a dark purple phenotype with thinner branches compared to C1. The origin of this colony was not precisely established. Colony 3 (C3) displayed a light brown to light pink phenotype with similar morphology to C2 and it was collected in the Northern Red Sea (D Allemand, 2016, personal communication), and it was classified as belonging to clade 4 (Keshavmurthy et al., 2013). All colonies used in this study have been kept in aquaculture for >10 years in recirculating systems. For the treatments applied in this study, coral colonies were initially acclimated under 200 ± 15 μmol photons m^−2^ s^−1^ in a 12:12 h light:dark cycle until colony pigmentation was visually stable (∼40 days). Water parameters were maintained at the following levels: salinity 35.0 ± 0.1 ‰, temperature see Fig. 1, pH 8.2 ± 0.2, nitrate-N 0.01 ± 0.01 mg L^−1^, phosphate-P 0.01 ± 0.01 mg L^−1^, calcium 420 ± 20 mg L^−1^, alkalinity 2.5 ± 0.2 mEq L^−1^. Constant water flow (10,500 L h^−1^) was provided by Voyager HP7 stream pumps (SICCE, Pozzoleone, Italy). The light was provided by pr-156W’s LED lamps (Orphek, Fairmount, IN, USA). This system provides light from the PAR spectrum exclusively (Fig. S1). To set up the experiment, each parent colony was cut down to 40 ramets and mounted on special CaCO_3_ breeding rocks (Fauna Marin, Holzgerlingen, Germany) numbered from 1 to 120 (ramet ID). High technical replication (*n* = 3 per colony) was chosen over higher true replication (*n* = 3) in order to test for intraspecific and intracolonial differences. All ramets were left to acclimate (under 200 ± 15 μmol photons m^−2^ s^−1^) and heal from fragmentation wounds for an additional 28 days before initial measurements, i.e., in total, colonies have been acclimated to 200 ± 15 μmol photons m^−2^ s^−1^ for 68 days in order to standardize pre-conditions. At day 0, ramets were evenly and randomly divided into two treatments, the “control treatment” (CT) and the “experimental treatment” (ET) (*n*_total_ = 120, *n*_treatment_ = 60, *n*_group_ = 20), thus encompassing six experimental groups, as follows: CT colony 1, 2, and 3 (CTC1, CTC2, and CTC3) and ET colony 1, 2, and 3 (ETC1, ETC2, and ETC3). The complete experiment run for a total of 68 days.

The CT and ET were both exposed to a light treatment in a 12:12 h light:dark cycle and exposed to artificially induced heat stress (Fig. 1). The CT was exposed to 200 ± 15 μmol photons m^−2^ s^−1^ (total daily dose of 8.6 ± 0.7 mol photons m^−2^) throughout the whole experiment (day 0–68) (Fig. 1). The ET was exposed to an elevated irradiance dose for 31 days (day 2–33) with a peak at 400 ± 20 μmol photons m^−2^ s^−1^ (total daily dose of 17.3 ± 0.9 mol photons m^−2^), eventually returning to 200 ± 15 μmol photons m^−2^ s^−1^ at day 33 (Fig. 1). The total dose of 17.3 ± 0.9 mol photons m^−2^ is similar to values measured in situ and it consists of a dose high enough to enhance thermal tolerance of *Coelastrea aspera* (Brown et al., 2002a). Based on the photoacclimation dynamics of *S. pistillata*, which exhibits changes in photopigment concentration and zooxanthellae densities within a time frame of several weeks (Titlyanov et al., 2001), we decided for an exposure to elevated irradiance for a 31-day period. At day 37, equal heat stress was induced on both light treatments by increasing temperature ∼0.5 °C d^−1^ until day 54 to a maximum of 33.4 ± 0.3 °C. This temperature was then kept relatively constant for seven days. Light measurements were done using a LI-193 spherical underwater quantum sensor (Li-Cor, Lincoln, NE, USA). Water temperature (ambient temperature of 25.8 ± 0.7 °C) was measured using HOBO data loggers (Onset Computer Corporation, Pocasset, MA, USA) every 30 min. Corals were not fed before or during the time of the experiment.

The entire experiment was performed in a single water column. While this strategy results in pseudoreplicates of the ramets (Hurlbert, 1984), we considered it as an option to avoid undesirable differences owed to tank effects and/or differences in water quality. Furthermore, the low accumulated biomass of the ramets (72 g at start, 101 g after light treatments), the constant monitoring and adjusting of water quality of the 500 L system, and the constant high water flow collectively prevent ramets from having any significant influence on the water quality (Putnam & Edmunds, 2011). To minimize potential tank effects, all ramets were spatially randomized semiweekly within their respective light treatment (i.e., the first 33 days). After day 33 (when the irradiance was uniform), the ramets were spatially randomized semiweekly between both treatments.

### Coral characteristics

#### Growth rates

To determine the potential effects of irradiance on coral growth rates, drip-dry weights were determined at the beginning of the experiment and after the ET ended (Wijgerde, Henkemans & Osinga, 2012). All ramets (*n* = 120) were weighed before and after being mounted on the breeding rocks in order to obtain the net and the total weight, and the combined weight for breeding rock/glue. At the end of the growth interval, the total weight of remaining ramets were corrected for the combined weight of the culture rocks and glue to obtain the net weight values. CT growth rates per colony (based on growth from the moment of fragmentation until the final weighing) were used to calculate the weight of all ramets at day 2. These data were used to calculate SGRs during the light treatment. Biofouling and water retention of the breeding rocks can occasionally cause an overestimation of growth, but, in this case, they were assumed to be equal among treatments (i.e., after normalization of the weighing procedure). The SGR for each individual was calculated as follows:

$${\rm{SGR}}({\rm{da}}{{\rm{y}}^{-1}}) = {{{\rm{ln}}{W_t}-{\rm{ln}}{W_{t-1}}} \over {\Delta t}}$$

where *W_t_* and *W_t_*_−1_ are the final and initial coral net weights, respectively, expressed in grams (g); Δ*t* is the growth interval in days. SGR is expressed in gram of coral per gram of coral per day, which can be simplified as day^−1^ (Wijgerde, Henkemans & Osinga, 2012).

A suite of factors is known to influence the growth of scleractinian corals, e.g., water flow and light intensity. Thus, we optimized our experimental setup to minimize potential influential factors, such as undesirable effects of different water flow patterns (referred to as “tank effects”). While effects of elevated irradiance include increased net photosynthetic and calcification rates (Moya et al., 2006; Schutter et al., 2008), increased water flow can also account for increased growth rates (Jokiel, 1978; Schutter et al., 2010, 2011). However, the latter does not result in photoinhibition of the zooxanthellae. Even though enhanced photosynthesis is not directly proportional to coral growth (i.e., calcification), a positive correlation between irradiance and calcification has been established in previous studies (Reynaud-Vaganay et al., 2001; Moya et al., 2006; Schlacher, Stark & Fischer, 2007; Cohen, Dubinsky & Erez, 2016).

#### Photographic analysis

A total of 18 ramets, three ramets per group (repeated) were randomly selected and photographed weekly throughout the course of the experiment. Coral ramets were placed into a small tank with fixed lighting and coral placement for normalization of the weekly procedure. Ramets were photographed using a tripod and a digital EOS 60D camera (Canon Inc., Tokyo, Japan) with fixed settings. Two photographs were taken per ramet and analyzed with Adobe Photoshop CC 2015 using luminosity (i.e., brightness), as a proxy for bleaching, in the histogram function (white equals a luminosity value of 255 and black equals the value of 0). The live tissue was selected using the “lasso tool”. Mean luminosity of both pictures was averaged and used for further analysis. Small differences were expected due to the tilt of the coral ramets in the small tank (i.e., shading), polyp expansion, coral specific pigmentation, and the angle and zoom of the digital camera. Luminosity values were recalculated to percentages, inverted, averaged per group, and renamed “Inverted Luminosity”, i.e., the value of 100 represents black and a value of 0 represents white; for reference: inverted luminosities of bleached or bleaching corals are closer to 0.

Moreover, photographs were analyzed for necrosis or “tissue loss” with Adobe Photoshop CC 2015 using the “lasso tool”. First, the entire ramet was selected (i.e., entire skeleton) and total pixels were counted using the histogram function. Subsequently, the selection was subtracted by deselecting tissue-free skeleton. The difference in total pixels selected was calculated to percentages and used for further analysis. Necrosis was diagnosed with the loss of coral tissue with clearly visible septa and corallites devoid of polyps.

### *Symbiodinium* characteristics

Zooxanthellae density and chlorophyll *a* were sampled four times during the experiment (Fig. 1). Three ramets per group were randomly selected for each sampling time (total *n* = 18). The first sampling point was set at day 1, shortly before the light treatment started. The second sampling point was set at day 24, at the peak of the ET. The third sampling point was set at day 36, shortly after the ET ended, and the fourth sampling point was set at day 56, at the heat stress highest value. Samples were collected by carefully cutting the ramets from the breeding rocks avoiding coralline and filamentous algae.

Coral tissue and zooxanthellae were separated from the coral skeleton using pressurized air (Loh et al., 2001) and suspended in 10 mL artificial seawater (35 ‰). Subsequently, the denser zooxanthellae were separated from the coral tissue by centrifugation at ∼4,200 rcf for 10 min. The resulting pellet was resuspended in 750 μL of artificial seawater (35 ‰).

#### Cell density and chlorophyll a

Zooxanthellae cells were counted immediately after sample collection using a Neubauer hemocytometer, and normalized to the coral surface area. Surface area was obtained by using the single wax dipping method, according to Veal et al. (2010). For the calibration curve, we used 15 spheres ranging from ∼1.0 to 18.2 cm^2^. The wax dipping was conducted using paraffin wax (Lamers & Indemans BV, ’s-Hertogenbosch, The Netherlands) and heated to 65 °C using an MGW Lauda MT thermostat (Beun de Ronde BV, La Abcoude, The Netherlands). Coral skeletons and calibration objects were kept at room temperature and weighed before and after wax dipping using an EMB 600-2 digital scale (Kern, Balingen, Germany) (accuracy of 0.01 g).

For chlorophyll *a* measurements, we added 1.35 mL of 90% acetone to a 0.15 mL zooxanthellae suspension with known cell count and stored samples in the dark at −20 °C for 48 h (after Welschmeyer, 1994). Samples were centrifuged for 10 min at 18,000 rcf. Chlorophyll *a* concentrations were measured using 1.2 mL of supernatant with a Trilogy Laboratory Fluorometer (Turner Designs, San Jose, CA, USA), and further normalized per zooxanthella cell.

#### Variable chlorophyll fluorescence

Effective quantum yield (Δ*F/Fm′*; light adapted) of PSII was measured with PAM fluorometry using a Mini-PAM (Walz GmbH, Effeltrich, Germany) with the following settings: actinic light OFF, saturation width 1.2 s, saturation intensity 12, damping 2, output gain 6 and measuring light 4, and all other settings kept on default. The EQY of eight ramets per group (repeated measurements) was obtained every two to three days during the light and heat stress treatments. We used the average of four spatially separated measurements per ramet. The fiber optic probe was positioned perpendicular to the ramet at a distance of ∼4 mm. Measurements were taken at set points (i.e., ∼5 h after lights turned on), in order to avoid differences due to circadian rhythms (Sorek & Levy, 2012).

#### Molecular analysis

Zooxanthellae samples collected at day 1 and day 36 were used to assess community composition over time by denaturing gradient gel electrophoresis (DGGE) analysis. For total DNA extraction, we used a phenol/chloroform protocol combined with CHAOS lysis-buffer (Fukami et al., 2004). A touchdown PCR was applied to amplify the ITS2 region of the zooxanthellae. This hypervariable region is located between the 5.8S and 28S region of the ribosomal RNA. Our approach amplified a DNA fragment ranging from 330 to 360 bp. For the PCR amplification, the following primer set was used: forward primer “ITSintfor2” and reverse primer “ITS2CLAMP” (LaJeunesse & Trench, 2000), with a clamp-GC in the reverse primer.

The PCR mixture contained 1× Bioline buffer 200 μM dNTPs, 2.5 mM MgCl_2_, 20 mg mL^−1^ bovine serum albumin, 0.5 μM reverse primer, 0.5 μM forward primer, 1.5 U Taq polymerase (Bioline, London, UK), 10 ng of DNA template, and sterile MilliQ water. The specificity of the amplification was increased by applying a “touchdown” protocol (Don et al., 1991). The thermal cycler protocol was 94 °C for 5 min, 20 cycles with the initial annealing temperature of 62 °C and decreasing 1 °C every two cycles until 52 °C. Additional 15 cycles were carried out using the annealing temperature of 52 °C. Each cycle consisted of 94 °C for 45 s, 62 to 52 °C for 45 s followed by 60 s at 72 °C, with a final extension at 72 °C for 10 min. The amplification products were checked for quality and quantity using a 1.5% agarose gel electrophoresis in 1× TAE buffer.

Equal volumes of PCR products were loaded onto an 8% polyacrylamide gel with a denaturing gradient ranging from 30% to 55% (the 100% denaturing solution contained 7 M urea and 40% formamide). Electrophoresis was performed in 0.5× Tris-acetate-ethylene diamine tetraacetic acid buffer using an Ingeny PhorU apparatus (Ingeny, Goes, The Netherlands). The gels were run for 16 h at 60 °C and 100 V, stained with Sybr Gold (Molecular Probes, Eugene, OR, USA), and further visualized and photographed under UV light.

### Statistical analyses

The statistical analyses were performed using the R version 3.3.2 (R Development Core Team, 2016). SGR, inverted luminosity (bleaching), necrosis (tissue loss), zooxanthellae density, and chlorophyll *a* concentrations, were analyzed with factorial analysis of variance (ANOVA) models, using *R*’s lm function. Interactions and main effects were tested with regular *F*-tests, using *R*’s anova function. The EQY was analyzed with a linear mixed model, using the lmer function from the lme4 package version 1.1-12 (Bates et al., 2015). Colony and ramet ID were used as random factors, light treatment and day as fixed factors. Significance tests for fixed and random effects were performed with the lmerTest package version 2.0-33 (Kuznetsova, Brockhoff & Christensen, 2016); *F*-tests with Satterthwaite approximation for denominator degrees of freedom were used for fixed effects and likelihood ratio tests for random effects. Final models were selected by backward elimination from full initial models, starting with the random part of the models. For random effects, the initial model consisted of ramet ID + light*colony, to allow for random colony by treatment effects; for fixed effects, the initial model included the light treatment by day interaction. The standard assumptions of homogeneity of variances and normality were checked by visual inspection of residual plots. Whenever significant departures from assumptions were observed for SGRs, zooxanthellae density or chlorophyll *a* concentrations, a log-transformation or double square root transformation of the response variable was sufficient to restore homogeneity and normality. The remaining parameters (bleaching, necrosis, EQY) were arcsine square root transformed to improve normality and homogeneity of residual variances. Post hoc multiple comparison tests with error-adjusted *p*-values were carried out using the multcomp package (Hothorn, Bretz & Westfall, 2008).

In addition, for each individual parameter, data were pooled using the average response per colony (*n* = 3) for further analyses. SGR was analyzed using a paired *t*-test (Wilcoxon signed-rank). Remaining parameters were analyzed using ANOVA, as described above, excluding ramet ID and colony as (random) factors.

Variations in the community composition of zooxanthellae obtained by DGGE were analyzed using the GelComparII software (version 6.5; Applied Maths, Sint-Martens-Latem, Belgium). The DGGE patterns were compared by creating a presence/absence matrix of all visual bands. Community composition similarity calculations between samples were conducted using PRIMER-E (version 6; PRIMER-E Ltd, Plymouth, UK). A non-metric multidimensional scaling (nMDS) plot was generated based on band profiles using the Jaccard distance. The analysis of similarities (ANOSIM) and permutational multivariate analysis of variance were performed based on the similarity calculations, in order to test for differences between colonies, treatments, and sampling days. The ANOSIM global *R* was calculated as the difference of between-tested-groups (i.e., colony, treatment or sampling day) and within-tested-groups mean rank similarities, and thus it displays the degree of separation between them. Complete separation is indicated by *R* = 1, whereas *R* = 0 suggests no separation (Clarke & Gorley, 2006).

## Results

### Coral characteristics

#### Growth rates

**Figure 2:**
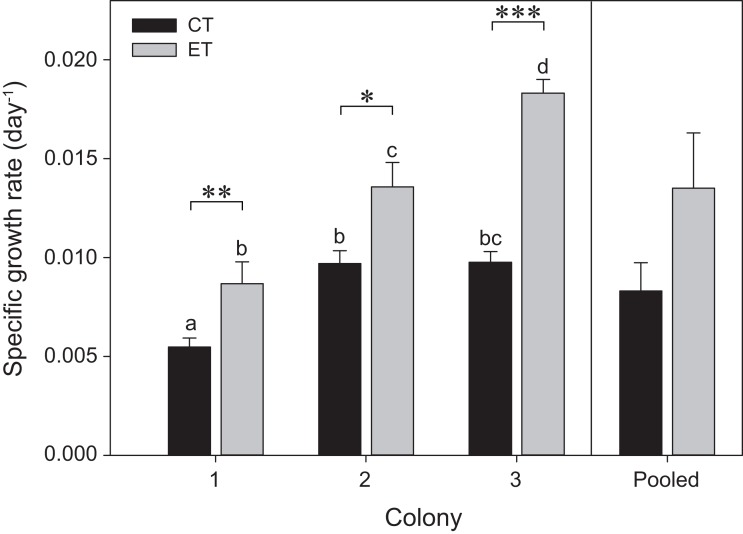
Specific growth rates for the three colonies of *S. pistillata* under control treatment (CT) and experimental treatment (ET) over a 35-day interval (i.e., before heat stress). Values are mean ± s.e. (*n* = 11 per colony, *n* = 3 for the pooled data). *Significant differences between light treatments (*p < 0.05, **p < 0.01, ***p < 0.001). Different letters indicate significant differences between groups.

The three colonies showed growth under both light treatments during the initial 35 days of the experiment (i.e., before heat stress) (Fig. 2). Significant effects of the factors “colony” and “light treatment” on coral growth rates were found (*p* < 0.001; Table S1). In general, corals of each colony exposed to the ET showed a significantly higher growth rate compared to those exposed to the CT, with C3 exhibiting a more pronounced increase in SGR comparatively to C1 and C2. No differences were found between light treatments for the pooled data analysis (*p* = 0.25; Fig. 2).

#### Photographic analysis

The three colonies showed bleaching and necrosis during heat stress. However, the degree of bleaching and necrosis differed significantly between colonies (*p* < 0.001; Table S1). While a significant effect of the factor “light treatment” was found for necrosis (*p* < 0.001; Table S1), no significant difference was found for bleaching (*p* = 0.097). The effect of light on necrosis differed between colonies as a significant interactive effect was found for the factors “colony” and “light treatment” (*p* < 0.001; Table S1).

**Figure 3:**
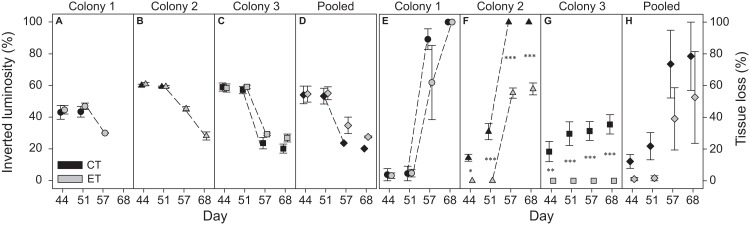
Bleaching (inverted luminosity) and necrosis (tissue loss) for the three colonies of *S. pistillata* during heat stress (data based on weekly photographs). (A) Bleaching colony 1; (B) bleaching colony 2; (C) bleaching colony 3; (D) bleaching pooled data; (E) necrosis colony 1; (F) necrosis colony 2; (G) necrosis colony 3; and (H) necrosis pooled data. Black symbols = control treatment (CT); gray symbols = experimental treatment (ET). Values are mean ± s.e. (*n* = 3). Dashed lines represent significant differences between successive data points (*p* < 0.05). *Significant differences between light treatments (*p < 0.05, **p < 0.01, ***p < 0.001).

Colony 1 displayed a consistently lower inverted luminosity comparatively to C2 and C3 (Figs. 3A–3C; Fig. S2). The inverted luminosity decreased in all groups after day 51, i.e., all ramets experienced bleaching. However, the inverted luminosity of CT ramets in C1 and C2 was not measured reliably after day 51 due to severe necrosis and eventually death of the ramets (Figs. 3E and 3F; Fig. S2). No C1 ramets survived the heat stress. ET subjected ramets of C2 experienced necrosis that stagnated after day 57 (Fig. 3F; Fig. S2). The CT subjected ramets experienced necrosis seven days prior to ET subjected ramets, and all ramets died after day 51 (Fig. 3F; Fig. S2). The ET subjected ramets of C2 survived the heat stress. In C3, only the CT subjected ramets experienced necrosis (Fig. 3G; Fig. S2). Necrosis stagnated after day 44 and the remaining tissue experienced bleaching. The ET subjected ramets showed no visible signs of necrosis (Fig. 3G; Fig. S2). The ramets of C3 survived heat stress. In general, necrosis was significantly more pronounced for CT ramets comparatively to the ET throughout the heat stress treatment (considering both C2 and C3) (Fig. 3; Fig. S2). A visual qualitative analysis of the remaining non-photographed ramets corroborated with the pattern observed in the photographed ramets along the experiment.

In contrast to the individual colonies, the pooled bleaching data revealed a significant decrease in inverted luminosity after day 51 only for the CT (*p* < 0.001). While significant effects were found for the factors “day” and “light treatment” in the pooled necrosis data (Table S2), no differences were found between subsequent data points or between light treatments per day.

### *Symbiodinium* characteristics

#### Cell density and chlorophyll a

**Figure 4:**
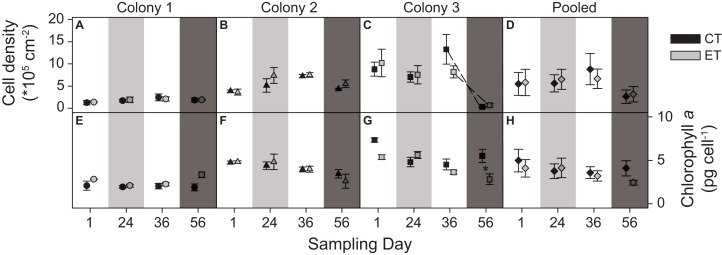
Zooxanthellae cell density (*10^5^) per cm^2^ and chlorophyll *a* content (pg) per zooxanthellate cell for the three colonies of *S. pistillata* at four sampling times (days). (A) Cell density of colony 1; (B) cell density of colony 2; (C) cell density of colony 3; (D) cell density of the pooled data; (E) chlorophyll *a* of colony 1; (F) chlorophyll *a* of colony 2; (G) chlorophyll *a* of colony 3; and (H) chlorophyll *a* of the pooled data. Black symbols = control treatment (CT); gray symbols = experimental treatment (ET). Values are mean ± s.e. (*n* = 3). Dashed lines represent significant differences between successive data points (*p* < 0.05). *Significant differences between light treatments (*p* < 0.05). Day 1 = start; day 24 = light treatment (light gray background); day 36 = between light treatment and heat stress; day 56 = heat stress (dark gray background).

The three colonies showed no significant differences in zooxanthellae density between light treatments. However, a significant interactive effect between the factors “day” and “colony” was found (*p* < 0.001; Table S1). This was evidenced by the different patterns in zooxanthellae densities between colonies over the course of the experiment. C1 exhibited consistently low zooxanthellae abundance throughout the experiment (Fig. 4A). In contrast, C2 had a higher zooxanthellae abundance throughout the light treatment and during the heat stress (Fig. 4B), while the zooxanthellae density of C3 showed the highest abundance at the beginning of the experiment, later decreasing strongly during the heat stress for both light treatments (sampling day 56; *p* < 0.001; Fig. 4C). No significant main effect or interactions were found in the pooled data analysis (Fig. 4D).

The light treatment significantly modified the effect of temperature on the concentration of chlorophyll *a*, as an interactive effect of the factors “day” and “light treatment” was found (*p* = 0.003; Table S1). C1 and C2 were not affected by the light treatment or the heat stress (Figs. 4E and 4F), while for C3, the pre-exposure to elevated light decreased chlorophyll *a* concentrations during the heat stress compared to the CT (sampling day 56; *p* = 0.003; Fig. 4G). No significant main effect or interactions were found in the pooled data analysis (Fig. 4H).

#### Variable chlorophyll fluorescence

**Figure 5:**
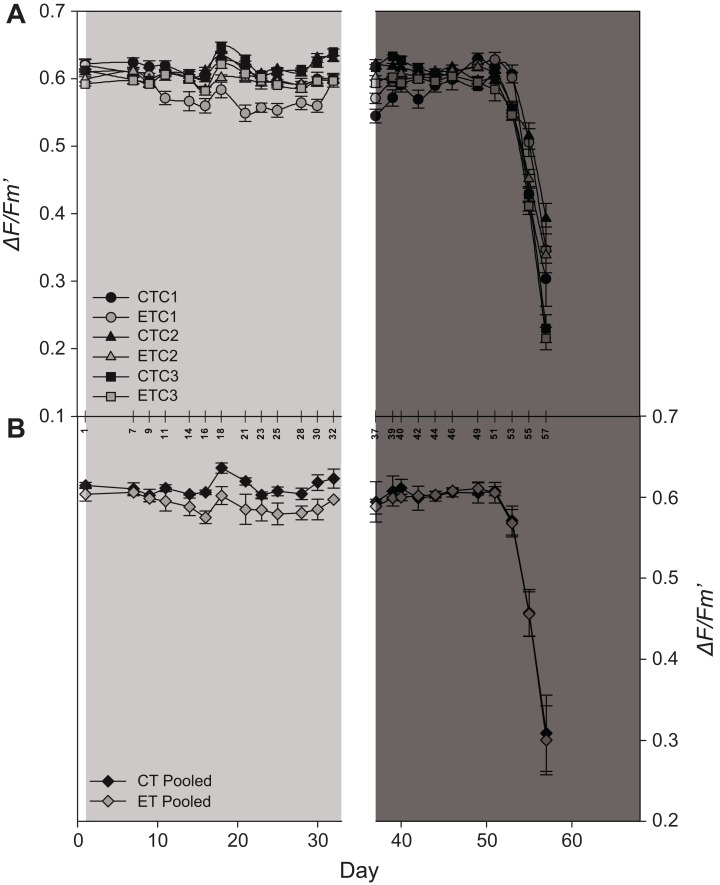
Effective quantum yield (*ΔF/Fm′*) of *S. pistillata* during the course of the experiment (A) per group (*n* = 8) and (B) pooled data (*n* = 3). Black symbols = control treatment (CT); gray symbols = experimental treatment (ET). Colony 1 (C1), 2 (C2), and 3 (C3). The 90° angled numbers correspond to the exact day at which EQY measurements were taken. Values are mean ± s.e.; day 2–33: light treatment (light gray background); day 37–68: heat stress (dark gray background).

Light had a significant effect on EQY during the first 32 days (i.e., during the light treatment) of the experiment (*p* < 0.001; Table S1). Even though a significant effect of the factor “colony” was found during the light treatment (*p* = 0.02; Table S1), in general, the ET exposed ramets displayed a lower EQY compared to the CT exposed ramets (Fig. 5A). No effect of light was found during heat stress (day 37–57). Also, significant effects were found for the factor “day” during both treatments (*p* < 0.001; Table S1; Fig. 5A). After day 51, EQY of all colonies strongly declined for both light treatments (Fig. 5A). Similar to the individual colonies, a significant effect of light was found during the first period in pooled data analysis, while in the second period only an effect of the factor “day” was found (Table S2; Fig. 5B).

#### Molecular analysis

**Figure 6:**
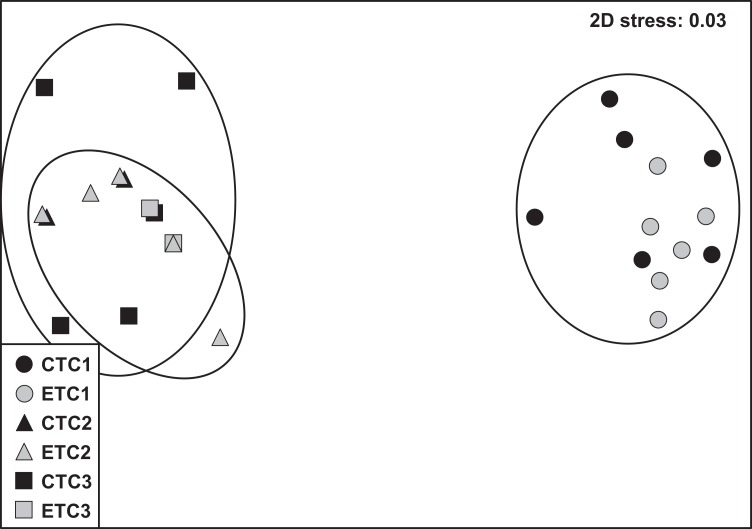
Non-metric multidimensional scale (nMDS) of zooxanthellae community per group based on PCR-DGGE band profiles using the Jaccard distance (*n* = 6). The NMDS plot displays samples according to light treatment (control [CT] or experimental [ET]) and colony (C1, C2, and C3).

Within each colony, the community composition of zooxanthellae did not change significantly over time. However, we found zooxanthellae community composition to differ significantly across different colonies (*p* = 0.001; Table S1). The analysis of similarity revealed that zooxanthellae community composition of C2 and C3 were more similar to each other than when compared to C1 (C1–C2: *R* = 1.000; C1–C3: *R* = 0.997; C2–C3: *R* = 0.406; see also Fig. S3). No significant differences were found for the factors “light treatment” and “day” within each colony. The nMDS plot displays the detailed clusters and description of the samples (Fig. 6).

## Discussion

Susceptibility of hard corals to thermal stress can vary significantly between species (interspecific; Baird et al., 2009; Kavousi et al., 2015), within species (intraspecific; Sotka & Thacker, 2005; Kavousi et al., 2015), and even within the same colony (intracolonial; Brown et al., 2002a, 2002b). The present study tested thermal stress responses based on recent light history using three colonies of the scleractinian coral *S. pistillata*. Distinct physiological (stress) responses between and within colonies revealed high intraspecific and intracolonial variability.

### Intraspecific and intracolonial variability

*Stylophora pistillata* is a species of hard coral with a wide-ranging spatial distribution that has resulted in four distinctly evolved clades (Keshavmurthy et al., 2013). Thus, experimental designs and outcome data are advocated to take into consideration these admissible clade differences. In the present study, we used two colonies of known clades (i.e., clade 1 for C1 and clade 4 for C3) and one unknown sample clade. However, based on the near indistinguishable zooxanthellae community (Fig. 6; Fig. S3) and the similar morphology of the C2 and C3 ramets, we assume C2 to be likely affiliated to clade 4.

**Figure 7:**
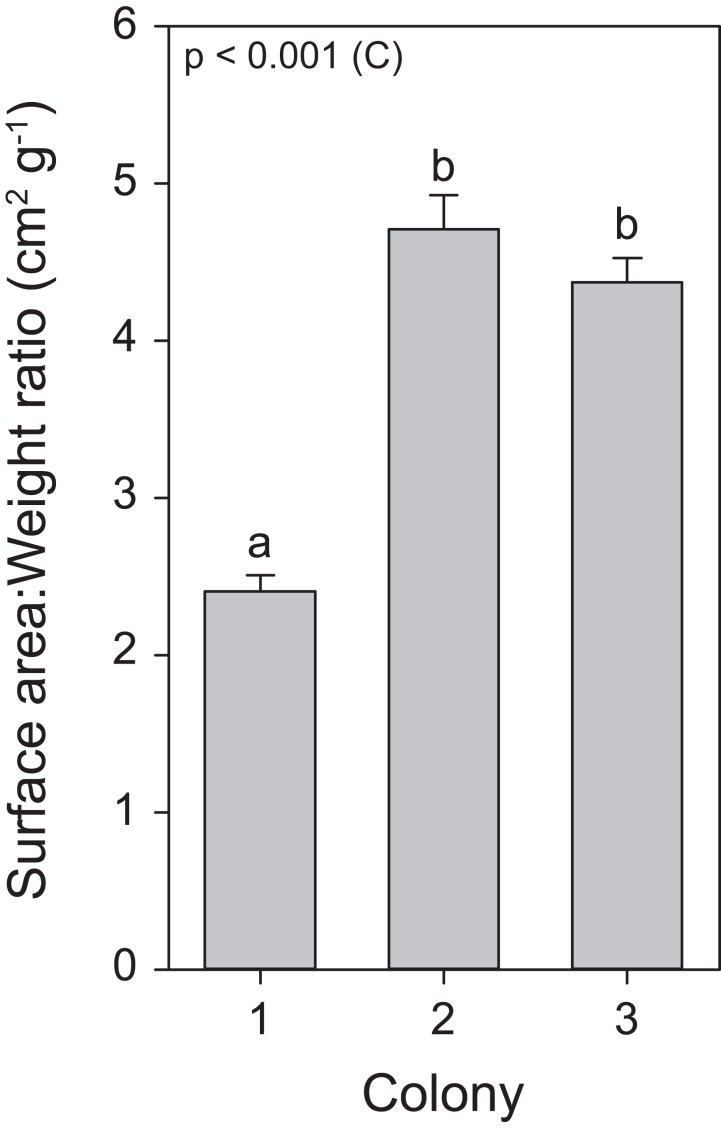
Surface area to weight ratio values for the three colonies of *S. pistillata*. Values are mean ± s.e. (*n* = 24). The significant factor (C = colony) is displayed based on one-way ANOVA (after log transformation). Different letters indicate significant differences between colonies based on a post hoc Tukey’s test (*p* < 0.001).

Throughout the course of the light treatment, all colonies showed significant growth, with C1 displaying the lowest rate. This can be explained by (or a combination of) three main features. First, as a reflection of the different locations and depths from which these colonies were originally collected, with possible adaptations to differential light environments that may reflect in their differing responses to exposed light intensities (Mass et al., 2007). Second, C1 had the lowest zooxanthellae density as well as chlorophyll *a* concentrations. The literature suggests that zooxanthellae positively affect calcification rates (Muller-Parker & D’Elia, 1997). Third, an analysis of surface area to weight ratio uncovered a significantly lower ratio for C1, in a similar range for *S. pistillata* previously reported by Hoegh-Guldberg (1988), i.e., 2.871 ± 0.074 (mean ± SE), while the ratio for C2 and C3 were equal to each other and higher than C1 (Fig. 7). In macroalgae, high surface area to volume ratios generally leads to elevated primary production (Rosenberg & Ramus, 1984; Gacia, Littler & Littler, 1996). Thus, the values of surface area to weight ratios in C2 and C3 may potentially lead to elevated primary production and subsequently increased calcification. To our knowledge, no study has yet correlated this ratio values to primary production of the zooxanthellae and calcification in scleractinian corals. In addition, in accordance with Osinga et al. (2011, 2012), all colonies displayed enhanced growth under elevated light exposure. As both zooxanthellae density and chlorophyll *a* concentrations remained equal within colonies in response to light before heat stress, the increased growth is likely to be attributed to light enhanced calcification (Mass et al., 2007; Cohen, Dubinsky & Erez, 2016).

While no effect of light treatment was found for inverted luminosity (i.e., bleaching) during heat stress, a significant effect for the factor “colony” was observed (Fig. 3). Here, C1 displayed the lowest zooxanthellae density and chlorophyll *a* concentrations along the whole experiment causing it to have a lighter color and subsequently a lower inverted luminosity. In addition, due to the degree of necrosis of each group, not every group was able to be properly analyzed in terms of inverted luminosity. C3 showed a significant decrease of zooxanthellae density on day 56, which was not followed by C1 and C2 (Fig. 3). However, all colonies experienced a significant reduction in inverted luminosity, and thus, evidencing the loss of zooxanthellae and/or photopigmentation at day 57 (Fig. 2).

In terms of necrosis and survival, for C2, the ET ramets survived while the CT ones died after 19 days in the heat stress treatment. In addition, the ET ramets of C3 showed no necrosis during the heat stress, in contrast to what was observed in the CT group. While all ramets of C1 died, a small portion of the ET ramets displayed delayed death compared to those from the CT. These intercolonial differences are possibly explained by (1) differential responses or starting densities/concentrations of both zooxanthellae and chlorophyll *a*; (2) the site where these colonies were originally collected; and/or (3) the orientation of the colony with respect to the direction of the light source. C3 was the first to expel its symbionts (also at the highest rate, see Fig. S2), and was the highest surviving colony. Even though overall EQY of the zooxanthellae during heat stress was similar among all groups, and despite bleaching being a negative response, this strategy ultimately may have prevented death from zooxanthellae derived reactive oxygen species and subsequent damage to coral host cells, especially in the elevated light exposed ramets (Lesser et al., 1990; Lesser & Farrell, 2004). In addition, in accordance with Brown et al. (2002a), the CT ramets of C3 also had higher chlorophyll *a* content per zooxanthella cell compared to ramets of the ET, which may have resulted in additional oxidative stress. However, C1 had both lowest zooxanthellae densities and chlorophyll *a* concentrations along the experiment, but reacted most negatively to heat stress that resulted in death of the ramets. As previously mentioned, EQY of all zooxanthellae during heat stress was similar, and chronic depression of the photosystems can be ruled out due to the lack of a light effect during heat stress. Thus, the difference in C1 is likely not caused by the zooxanthellae community but more likely due to the coral host and its history. Moreover, due to its high latitude location, the (Northern) Red Sea experiences strong fluctuations in environmental parameters (Cardini et al., 2015). Both C2 and C3 originated from the Red Sea, which is commonly known as a “refuge” for corals because of high thermal tolerance, also for *S. pistillata* (Fine, Gildor & Genin, 2013; Krueger et al., 2017). Corals exposed to highly variable conditions often exhibit increased thermal tolerance (Palumbi et al., 2014). Thus, both C2 and C3 may have had a historically acquired benefit, i.e., elevated thermal tolerance, compared to C1. Lastly, all colonies experienced necrosis but the necrosis development differed between C1 and the remaining two colonies. CTC2, ETC2, and CTC3 experienced necrosis from the base upwards while necrosis of C1 in both light treatments was apparently more stochastic. Attenuation of light is a logical result caused by the morphology of the coral (Kaniewska, Anthony & Hoegh-Guldberg, 2008; Kaniewska et al., 2011). While the incident downwelling radiation was similar within each treatment at the tip of all ramets, the thinner, longer, and vertically inclined branches of C2 and C3 (Fig. S2) toward the light source may have caused a heterogeneous light environment along the branch, resulting in higher incident radiation near the tip of the branches (Kaniewska et al., 2011). Subsequently, the coral tips received elevated PAR, and according to the hypothesis of IETT, this may have caused the survival of ET ramets from C2 in particular. In contrast, the thicker, (planar) less vertically inclined branches of C1 (Fig. S2) may have had allowed for a more homogeneous light environment causing a relatively even spread of necrosis.

#### Light as a modulator of thermal tolerance

Irradiance enhanced thermal tolerance has been established in *C. aspera* (Brown et al., 2000a, 2002a, 2002b), while an analogous result was found for four other scleractinian corals including *S. pistillata* (Ferrier-Pagès et al., 2007). In the present study, two out of the three colonies of *S. pistillata* revealed significantly enhanced thermal tolerance when pre-exposed to the experimental light treatment. Due to the significant difference in EQY observed during the light treatments and the increased growth rate under elevated irradiance, we suggest that irradiance was the main parameter responsible for the enhanced thermal tolerance (rather than undesirable “tank effects”, as for example, differences in water flow). Thus, enhanced thermal tolerance found in this study is likely IETT. However, the heat stress responses observed here are in contrast with those of Brown et al. (2002a). Our results are primarily based on observed necrotic responses, while those from Brown and colleagues are based on bleaching responses. We argue that several reasons may be accounting for these contrasting results, as follows: (1) The constant flux of available light. The constant exposure to 400 μmol photons m^−2^ s^−1^ for 12 h per day, resulted in a total irradiance dose of 17.3 mol photons m^−2^, which is similar to that observed in situ causing bleaching tolerance in *C. aspera* (Brown et al., 2002a). However, solar irradiance on coral reefs is characterized by fluctuating values on a (daily) spatio-temporal scale (see Wangpraseurt et al. (2014) and references therein). In addition, the IETT hypothesis assumes that due to exposure to higher PAR, the coral host protects itself against oxidative and light stresses by the synthesis of antioxidizing enzymes and (fluorescent) stress-related proteins (Brown et al., 2000b, 2002b; Brown & Cossins, 2011; D’Angelo et al., 2012; Hawkins et al., 2015). The EQY data indicated only a modest level of constant light stress, which may not be sufficient to chronically depress photosystems and induce adequate increased synthesis of protective antioxidants. Thus, enhanced bleaching tolerance may only be acquired by elevated total irradiance with temporary daily exposure to extreme irradiances (i.e., exceeding 1,000 μmol photons m^−2^ s^−1^); (2) The duration of the light treatment. Enhanced bleaching tolerance of *C. aspera* was obtained after elevated light exposure over several months (∼4.5 months; Brown et al., 2002a). Here, ramets were exposed to an elevated light treatment for a total of 31 days. Thus, the duration of the light treatment may have been inadequate to induce proper (photo)acclimation (Cohen & Dubinsky, 2015) and enhance bleaching tolerance; (3) The limited spectrum of available light. Ferrier-Pagès et al. (2007) were able to induce an analogous tolerance, i.e., a lack of photoinhibition, after 17 days of exposure to UVR (wavelengths not present in our light source, see Fig. S1) instead of increased PAR. Thus, the constant irradiance level, the duration of exposure and spectrum provided in our experiment is likely to explain the unobserved enhanced bleaching tolerance in the ET groups; (4) The thickness of coral tissue. In addition to irradiance, the differential responses, i.e., a bleaching versus necrotic response, can occur due to morphological differences between the thin-tissued *S. pistillata* and the thick-tissued *C. aspera* used by Brown et al. (2002a, 2002b). Wangpraseurt et al. (2012) found that light attenuation is more evident in thick-tissued corals than in thinner-tissued corals such as *S. pistillata*. Thus, it is likely that the latter is less efficient in performing photoprotection of its zooxanthellae (Salih, Hoegh-Guldberg & Cox, 1998; Dimond, Holzman & Bingham, 2012; Wangpraseurt et al., 2017), potentially ensuing an extra damage to the coral; and (5) Extreme temperatures. Lastly, corals in the present study were exposed to extreme temperatures reaching up to 33.7 °C, which is similar to temperatures used by Brown and colleagues (Brown et al., 2002a). *S. pistillata* is known to be more susceptible to thermal stress than *Coelastrea* (McClanahan et al., 2004; Baird et al., 2009). Even though we opted for slow heating of ∼0.5 °C d^−1^, the final temperature of 33.7 °C may have been too high to properly assess bleaching responses.

While some intraspecific differences described here can likely be explained by genotypic variation between zooxanthellae (i.e., resulting in differential photophysiology of the corals), intracolonial differences suggest that the coral host plays a significant role in thermal tolerance (sensu Brown et al., 2002b; Baird et al., 2009; Bellantuono, Hoegh-Guldberg & Rodriguez-Lanetty, 2012; Brown et al., 2015). Even though no changes in zooxanthellae community composition within colonies were evident through time, a full investigation, including the proper quantification of the relative abundances of zooxanthellae clades/types can largely contribute to our understanding of the physiological responses to thermal stress events, including those observed in this study. Moreover, it is worth mentioning that our study is limited by the low number of coral colonies used (*n* = 3), and that the use of DGGE did not allow for an in depth analysis of the potential effect of *Symbiodinium* genotypes on the photophysiological responses of coral hosts. Thus, we suggest that future studies should incorporate alternative approaches to more accurately access the community structure and abundance of *Symbiodinium* in each individual studied ramet (for instance, by using a combination of high-throughput sequencing and specific quantitative PCR assays), as well as expand the spectrum of coral genotypes investigated.

#### Intercolonial versus pooled data

In general, analysis of pooled data is not reliably representative for all the individual colonies tested (sensu Kavousi et al., 2015). Most importantly, in some cases, the use of pooled data can reveal a completely opposite outcome from what is obtained for the individual colony analysis. For instance, in this study, no significant increase in SGR was found under the elevated light in the pooled data, whereas the individual colonies clearly displayed light-enhanced calcification. In addition, pooled data on necrosis showed no differences between light treatments or subsequent days, whereas the former was observed in C2 and C3, and the latter in C1 and C2. Furthermore, differences found only in one of the colonies were masked by the pooled data analysis for both zooxanthellae density and chlorophyll *a*. No differences were found over time between subsequent days or light treatments, i.e., C1 and C2, while C3 behaved rather differently by showing decreased zooxanthellae densities at day 56 and differing chlorophyll *a* concentrations between light treatments during heat stress. In this study, only EQY data yielded similar results between the individual responses and pooled data analyses.

## Concluding Remarks

A number of studies have investigated the induction of thermal tolerance in scleractinian corals by pre-exposure treatments to elevated irradiances. Here, we designed a simplified microcosm experiment to investigate the induction of thermal tolerance at both intraspecific and intracolonial levels. We applied a combination of physiological measurements of corals in combination with the molecular assessment of their dinoflagellate symbiont. We show that colonies of *S. pistillata*, affiliated to clade 4 (Keshavmurthy et al., 2013), are likely to acquire a form of IETT that was evidenced by a less severe, delayed, and/or non-necrotic response in elevated light exposed ramets. In addition, the time-series data analysis enabled us to disentangle several factors that might account for these differential and time-dependent thermal stress responses of the colonies evaluated throughout the course of the experiment. Worth mentioning, we acknowledge that the limited number of colonies tested (*n* = 3) prevents our study to extrapolate ecologically relevant conclusions. However, we advocate our findings provide a rational basis for future prospective experimental design. Importantly, we posit that, although pooling data is a common practice in ecological and physiological studies to investigate populations, doing so when studying coral responses to light and heat-stress may lead to incorrect interpretations and/or inaccurate assumptions. We recommend that, in addition to population responses, a better understanding of physiological processes in reef-building scleractinian corals can be achieved by experimental designs encompassing a larger number of colonies and focusing on investigating colony-specific responses through the use of high replication units. In addition, in relation to testing the IETT, we recommend the use of in situ collected colonies, and a light environment more closely related to that described by Brown et al. (2002a, 2002b), by e.g., increasing duration of the light treatment and including UVR in the light spectrum.

## Supplemental Information

10.7717/peerj.3802/supp-1 Supplemental Information 1 Raw data.

10.7717/peerj.3802/supp-2 Supplemental Information 2 Figure S1: Image of light spectrum. Spectrum of Orphek PR-156W.

10.7717/peerj.3802/supp-3 Supplemental Information 3 Figure S2: Image of each group of ramets after fragging and during heat stress. Photographs of *Stylophora pistillata* ramet (one ramet per group) after fragging, and during heat stress (day 44, 51 and 57). Control treatment (CT). Experimental treatment (ET). Colony 1 (C1), Colony 2 (C2) and Colony 3 (C3).

10.7717/peerj.3802/supp-4 Supplemental Information 4 Figure S3: Image of DGGE gel (Symbiodinium ITS2). DGGE gel displaying Symbiodinium community based on ITS2.

10.7717/peerj.3802/supp-5 Supplemental Information 5 Table S1: Table displaying significant main effects and interactions (including colony). Summary of significant main effects and interactions based on factorial analysis of variance for specific growth rate (during light treatment), cell density, chlorophyll *a* and photographic analyses, linear mixed model for fluorescence (EQY) and permutational multivariate analysis of variance for zooxanthellae community.

10.7717/peerj.3802/supp-6 Supplemental Information 6 Table S2: Table displaying significant main effects and interactions (excluding colony). Summary of significant main effects and interactions based on factorial analysis of variance for photographic analyses and fluorescence (EQY).

## Additional Information and Declarations

### Data Availability

The following information was supplied regarding data availability: The raw data has been supplied as Supplemental Dataset Files.
