# Supplementary figures and images for: Light induced intraspecific variability in response to thermal stress in the hard coral Stylophora pistillata
Source: PeerJ. 2017 Oct 11;5:e3802. doi: 10.7717/peerj.3802 (PMC5640979; doi:10.7717/peerj.3802)

**AFTER  
FRAGGING**

**44**

**51**

**57**

**CTC1**

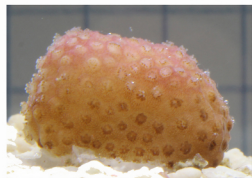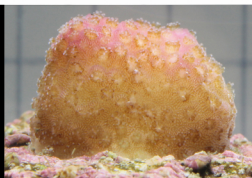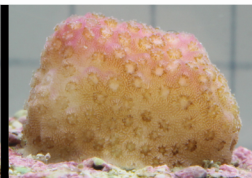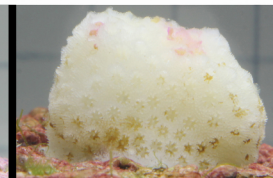

**ETC1**

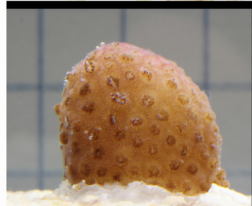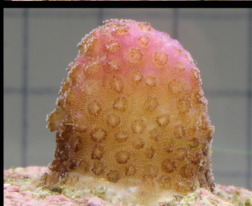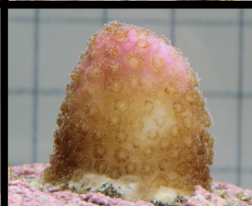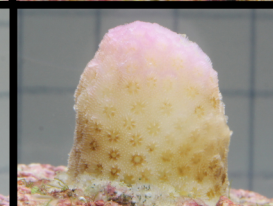

**CTC2**

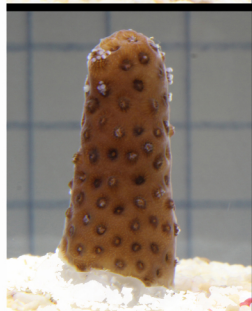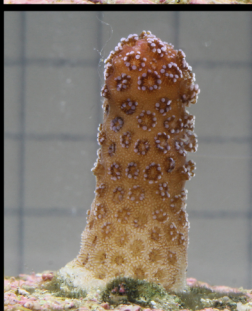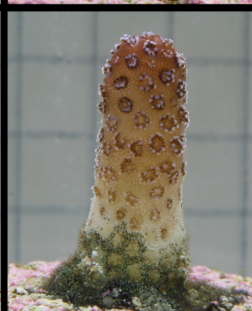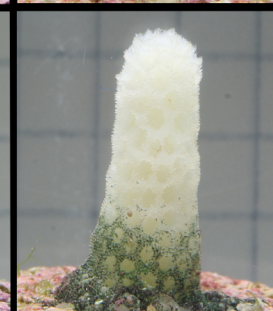

**ETC2**

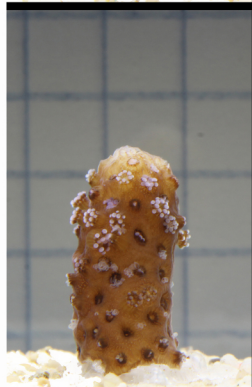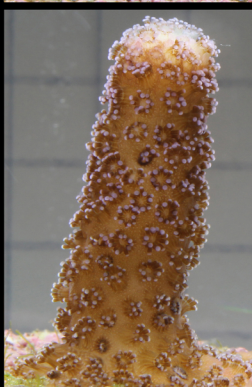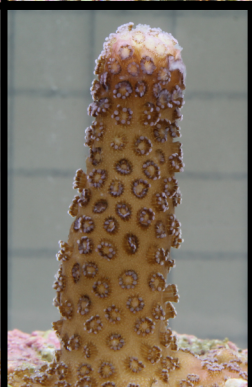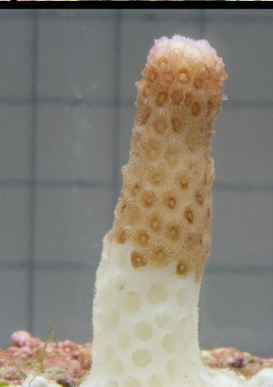

**CTC3**

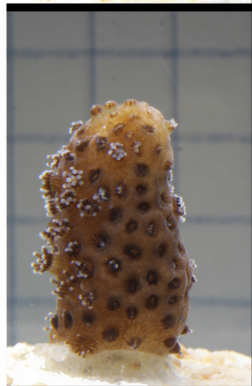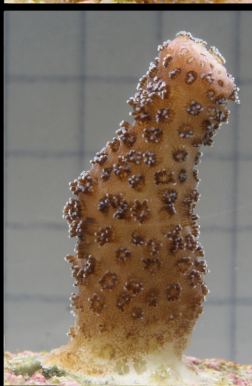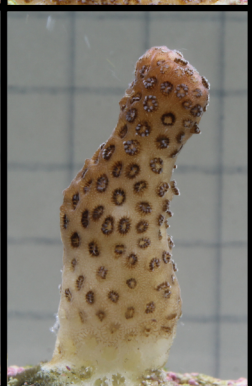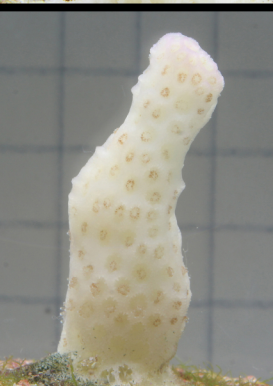

**ETC3**

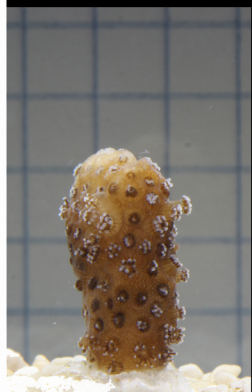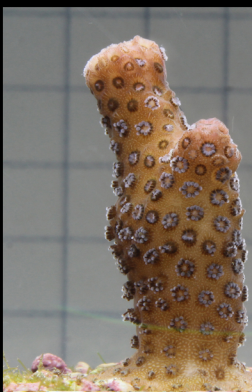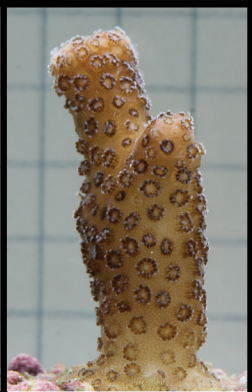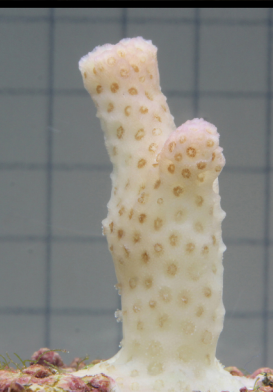

Supplement: Supplemental Information 3 — Photographs of Stylophora pistillata ramet (one ramet per group) after fragging, and during heat stress (day 44, 51 and 57). Control treatment (CT). Experimental treatment (ET). Colony 1 (C1), Colony 2 (C2) and Colony 3 (C3). [file peerj-05-3802-s003.pdf]

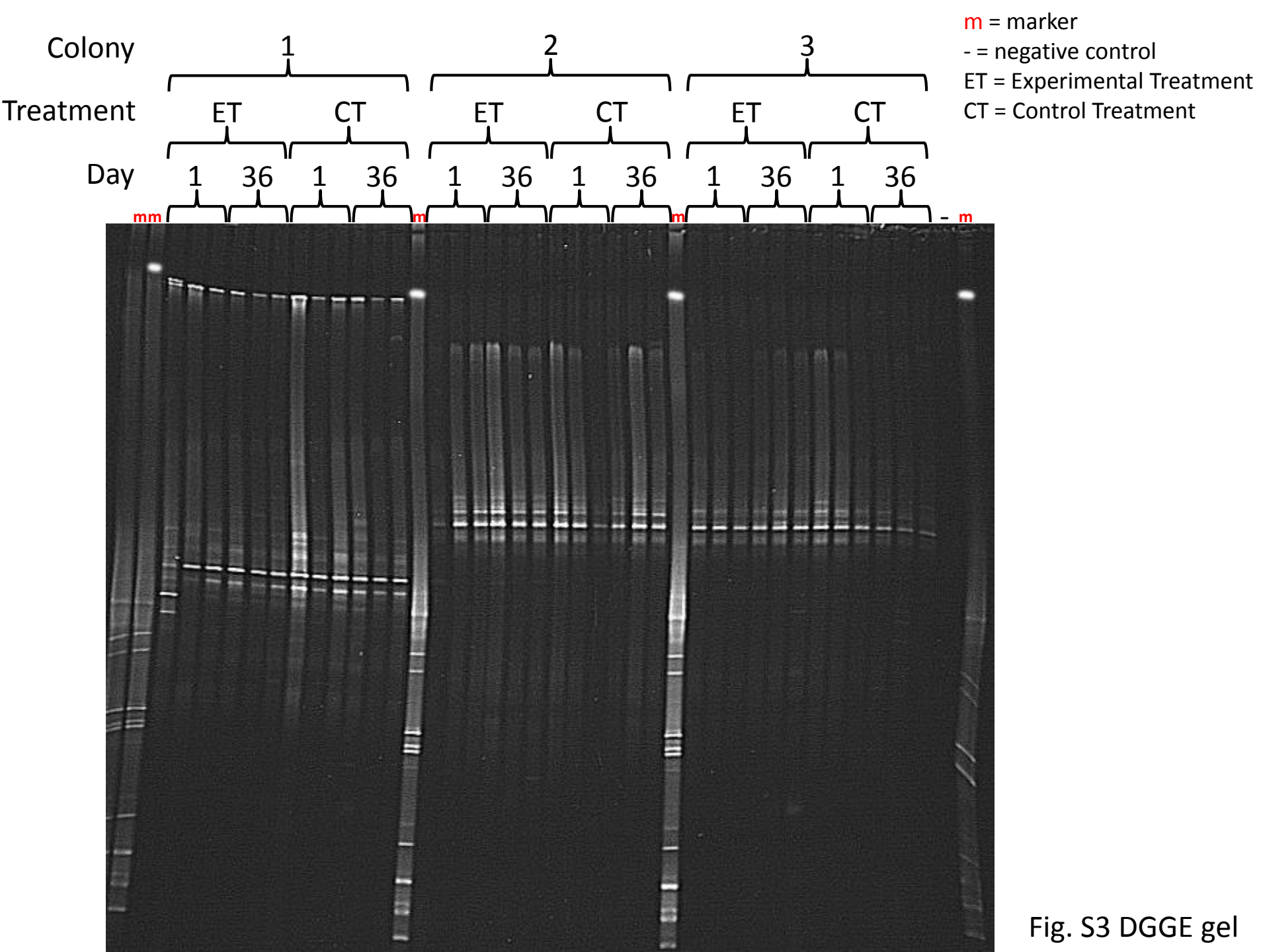

Fig. S3 DGGE gel

Supplement: Supplemental Information 4 — DGGE gel displaying Symbiodinium community based on ITS2. [file peerj-05-3802-s004.pdf]
