# Supplementary material for: Light induced intraspecific variability in response to thermal stress in the hard coral Stylophora pistillata
Source: PeerJ. 2017 Oct 11;5:e3802. doi: 10.7717/peerj.3802 (PMC5640979; doi:10.7717/peerj.3802)
Supplement: Supplemental Information 5 — Summary of significant main effects and interactions based on factorial analysis of variance for specific growth rate (during light treatment), cell density, chlorophyll a and photographic analyses, linear mixed model for fluorescence (EQY) and permutational multivariate analysis of variance for zooxanthellae community. [file peerj-05-3802-s005.docx]

**Table S1** Summary of significant main effects and interactions based on factorial analysis of variance for specific growth rate (during light treatment), cell density, chlorophyll *a* and photographic analyses, linear mixed model for fluorescence (EQY) and permutational multivariate analysis of variance for zooxanthellae community.

| Specific growth rate (during light treatment) | | | | | | | |
| --- | --- | --- | --- | --- | --- | --- | --- |
|  | df | | | *F* | | *p* | |
| Light Treatment | 1 | | | 53.9 | | <0.001 | |
| Colony | 2 | | | 41.6 | | <0.001 | |
| Photographic analysis (Inverted Luminosity) | | | | | | | |
|  | df | | | *F* | | *p* | |
| Day | 3 | | | 177.2 | | <0.001 | |
| Colony | 2 | | | 36.6 | | <0.001 | |
| Day:Colony | 5 | | | 6.5 | | <0.001 | |
| Photographic analysis (Necrosis/Tissue lost) | | | | | | | |
|  | df | | | *F* | | *p* | |
| Day | 3 | | | 158.7 | | <0.001 | |
| Light Treatment | 1 | | | 136.5 | | <0.001 | |
| Colony | 2 | | | 78.6 | | <0.001 | |
| Day:Light Treatment | 3 | | | 2.9 | | 0.044 | |
| Day:Colony | 6 | | | 37.1 | | <0.001 | |
| Light Treatment:Colony | 2 | | | 25.8 | | <0.001 | |
| Cell density | | | | | | | |
|  | df | | | *F* | *p* | | |
| Day | 3 | | | 21.9 | <0.001 | | |
| Colony | 2 | | | 40.9 | <0.001 | | |
| Day:Colony | 6 | | | 20.7 | <0.001 | | |
| Chlorophyll *a* | | | | | | | |
|  | df | | | *F* | *p* | | |
| Day | 3 | | | 9.6 | <0.001 | | |
| Light Treatment | 1 | | | 13.1 | <0.001 | | |
| Colony | 2 | | | 73.4 | <0.001 | | |
| Day:Light Treatment | 3 | | | 5.4 | 0.003 | | |
| Day:Colony | 6 | | | 4.3 | 0.002 | | |
| Variable chlorophyll fluorescence Day 1 - 32 | | | | | | | |
|  | df | | | *F* | *p* | | |
| Day | 12 | | | 10.1 | <0.001 | | |
| Light Treatment | 1 | | | 36.3 | <0.001 | | |
| Colony | - | | | - | 0.02 | | |
| Day:Light Treatment | 12 | | | 4.5 | <0.001 | | |
| Variable chlorophyll fluorescence Day 37 - 57 | | | | | | | |
|  | df | | | *F* | *p* | | |
| Day | 12 | | | 10.1 | <0.001 | | |
| Zooxanthellae community | | | | | | | |
|  | | df | Pseudo-*F* | | | | *p* |
| Colony | | 2 | 34.2 | | | | 0.001 |
