# Supplementary material for: Light induced intraspecific variability in response to thermal stress in the hard coral Stylophora pistillata
Source: PeerJ. 2017 Oct 11;5:e3802. doi: 10.7717/peerj.3802 (PMC5640979; doi:10.7717/peerj.3802)
Supplement: Supplemental Information 6 — Summary of significant main effects and interactions based on factorial analysis of variance for photographic analyses and fluorescence (EQY). [file peerj-05-3802-s006.docx]

**Table S2** Summary of significant main effects and interactions based on factorial analysis of variance for photographic analyses and fluorescence (EQY).

| Photographic analysis (Inverted Luminosity) | | | | |
| --- | --- | --- | --- | --- |
|  | df | *F* | | *p* |
| Day | 3 | 15.3 | | <0.001 |
| Photographic analysis (Necrosis/Tissue lost) | | | | |
|  | df | *F* | | *p* |
| Day | 3 | 5.6 | | 0.008 |
| Light Treatment | 1 | 136.5 | | 0.031 |
| Variable chlorophyll fluorescence Day 1 - 32 | | | | |
|  | df | *F* | *p* | |
| Light Treatment | 1 | 27.7 | <0.001 | |
| Variable chlorophyll fluorescence Day 37 - 57 | | | | |
|  | df | *F* | *p* | |
| Day | 10 | 24.8 | <0.001 | |
